# 3D printing of patient-specific neck splints for the treatment of post-burn neck contractures

**DOI:** 10.1186/s41038-018-0116-1

**Published:** 2018-06-08

**Authors:** Dafydd O. Visscher, Sjoerd te Slaa, Mariëlle E. Jaspers, Marloes van de Hulsbeek, Jorien Borst, Jan Wolff, Tymour Forouzanfar, Paul P. van Zuijlen

**Affiliations:** 1 Department of Plastic, Reconstructive and Hand Surgery, Amsterdam Movement Sciences, VU University Medical Center, de Boelelaan 1117, 1081HV Amsterdam, the Netherlands; 2 3D InnovationLab, VU University Medical Center, Amsterdam, the Netherlands; 3 Department of Oral and Maxillofacial Surgery/Oral Pathology, VU University Medical Center, Amsterdam, the Netherlands; 4 Department of Medical Technology, VU University Medical Center, Amsterdam, the Netherlands; 5 Department of Plastic, Reconstructive and Hand Surgery/Burn Center, Red Cross Hospital, Beverwijk, the Netherlands; 6 Department of Occupational Therapy, Red Cross Hospital, Beverwijk, the Netherlands

**Keywords:** Neck burns, Contracture, 3D printing, Neck splint, Clinical, Optical 3D scanning

Dear Editor,

Burn scar contracture is a common problem in healing burn wounds of the neck. It can cause both pain and dysfunction if not treated adequately [1]. The treatment of such wounds often involves a combination of surgery and splinting therapy [2]. A variety of splints, including the thermoplastic static neck splint [3], the Watusi collar [4], manually fabricated splints, and pre-fabricated splints such as the Philadelphia collar have been used for the management of scar contractures. However, each type of splint has its own advantages and disadvantages, and none of these splints seem to reduce the need for skin reconstruction nor delays the time until surgical reconstruction [5].

Medical applications for optical three-dimensional (3D) scanning and 3D printing are evolving rapidly [6], and both technologies could revolutionize the field of burn and wound care. More specifically, optical 3D scanners in combination with 3D printing technologies can be used to manufacture patient-specific devices such as splints for the treatment of post-burn neck contractures. The combination of these technologies could increase product customization, production speed, and cost-effectiveness of splint development. 3D printing is already being used for surgical planning, education, and implant customization [7]. Furthermore, facial masks have already been manufactured for burn patients using optical 3D scanning and 3D printing technologies [8, 9]. The aim of this study was to determine whether clinical use of these technologies for the production of patient-specific neck splints is feasible in a group of burn patients.

**Fig. 1:**
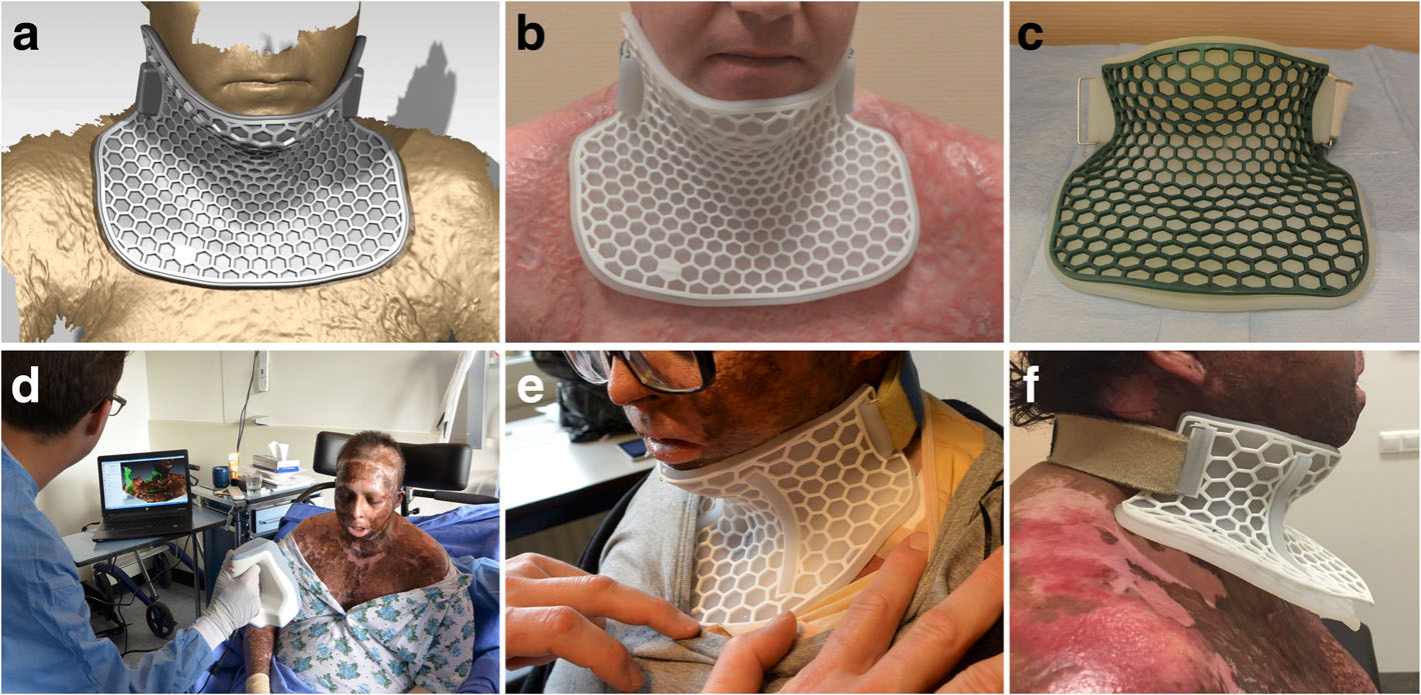
Design and three-dimensional (3D) printing of patient-specific splints for neck burns. **a** Computer-aided design of neck splint. **b** 3D-printed neck splint on the same patient. **c** 3D-printed neck splint in color (for patient 5). **d** Contactless optical 3D scanning process. **e** 3D-printed neck splint on the same patient. **f** three months after wearing neck splint (note anatomical change)

A retrospective study was performed with six patients who had been treated for burns and burn-related neck contractures at the Red Cross Hospital Burn Center in Beverwijk, the Netherlands. Following admission, all patients with neck burns were scanned using an optical 3D scanner (Artec Spider™; Artec Group, Moscow, Russia). Following scanning, all patients received a 3D-printed neck splint consisting of silicone and medical-grade nylon (Fig. 1a–c) instead of a standard neck splint (which in our burn center is a manually fabricated neck splint). In order to determine patient satisfaction, a telephonic questionnaire was administered to all patients (Table 1). The study was approved by the regional Medical Ethics Committee of Noord-Holland, the Netherlands (M016-004). In light of the Declaration of Helsinki, all patients gave oral informed consent to start the intervention. Additional written informed consent was obtained from patients whose photographs were used for publication.

**Table 1:** Patient demographics and response to questionnaire following wearing of three-dimensional (3D)-printed neck splint

Patient demographics						Patient response				
Patient	Age (years)	TBSAB (%)	Initiation of splint (days)	No. of splints received	Follow-up (months)	Comfortability (1–10)	Daytime wear (hours)	Nighttime wear	Suggestions	Overall grade
1 (M)	42	56.5	29	3	19	8.5	7	No	Strap	8.5
2 (F)	43	60	13	2	16	7	14	No	–	8
3 (M)	72	18	46	1	1	8	5	No	Strap	8
4 (F)	25	18	31	1	7	1	2	No	–	1
5 (M)	9	31	14	4	7	6.5	8	Yes	Strap	7.5
6 (M)	42	44.6	4	2	5	6.5	14	Yes	Strap	8
Median (Q1–Q3)	42 (21–50.3)	37.8 (18–57.4)	21.5 (10.8–34.8)	2 (1–3.3)	7 (4–16.8)	6.8 (5.1–8.1)	7.5 (4.3-14)	–	–	8 (5.9–8.1)

Daytime wear (hours) refers to the number of hours the patient wore the splint during the day. Nighttime wear refers to whether the patient wore the splint while sleeping.

Q1: 25th percentile, Q3: 75th percentile

*M* male, *F* female, *TBSAB* total body surface area burned

One engineer with in-depth knowledge in medical 3D scanning performed all optical 3D scans, including post-processing. Optical 3D scanning took 30 min, including setup (Fig. 1d). Computer-aided design was completed in approximately 4 h. 3D printing of the splints took approximately 4 days because not all the parts could not be printed in-house. Therefore, the total production time for one 3D-printed neck splint was 5 days. The thickness of the final silicone splints and overlying nylon honeycomb scaffolds were 5 and 3 mm respectively.

The median follow-up after the initiation of the 3D-printed splints was 7 months (interquartile range (IQR): 4–16.8) (Table 1). The comfort levels of the 3D-printed splints were satisfactory (6.8, IQR: 5.1-8.1). Patients wore their splints for long periods during the day (7.5h, IQR: 4.3-14h), but only two out of six patients wore their splints at night (during sleep). The splints were graded very well overall with a score of 8 out of a maximum 10 points (IQR: 5.9–8.1). Patients suggested to improve the splint straps since they caused itching and occasionally neck pain (Table 1, Fig. 1e–f). All splints fitted well and no major complications were observed.

In this study, we show that patient-specific neck splints can be fabricated using optical 3D scanning and 3D printing technology. With current advances in optical 3D scanning, computer-aided design, and 3D printing, patient-specific neck splints can be manufactured precisely and contactless; patients could be scanned at any given time, even in the operating room. The fabrication workflow took approximately 5 days to complete. Although this is faster than the manually fabricated splints (~ 7 days) at our burn center, many other splints can be fabricated much faster (e.g., Watusi collar and Philadelphia collar). In the near future, mobile phones with scanning software might be accurate enough to perform a scan of the neck area, which subsequently accelerates the production process [10].

The production costs for patient-specific neck splints are higher than for currently available splints. Therefore, clinical application of 3D-printed splints is, at this point, not yet cost-effective. If the costs could be reduced by using cheaper optical 3D scanners and free open source CAD software, patient-specific splints might become available for widespread clinical application in the near future.

One big advantage of using CAD software for modeling of splints is the ease of changing specific parameters during wound healing. Although some other splints can also be adjusted after anatomical change (e.g., thermoplastic splints), these always require patient contact to fit perfectly. In our study, most patients required additional splints due to anatomical changes during wound healing (Fig. 1f). This problem could be solved rapidly by designing a splint to fit the new anatomical situation. This iterative process optimizes patient treatment and could eventually contribute to the treatment of burns in remote areas. The authors believe that in the near future, parametric modeling may contribute to the development of bioinspired splints with pressure distribution and even local drug release.
